# Modulation of Pulmonary Inflammation and the Redox Pathway In Vitro and In Vivo by Fumaric Ester

**DOI:** 10.3390/antiox14091141

**Published:** 2025-09-22

**Authors:** Aline Pontes de Oliveira, Alexsandro Tavares Figueiredo-Junior, Priscilla Cristine de Oliveira Mineiro, Evelyn Caribé Mota, Carolinne Souza de Amorim, Helber da Maia Valenca, Aline Cristina Casimiro de Albuquerque Gomes, Sabrina Sodré de Souza Serra, Pedro Leme Silva, Christina Maeda Takiya, João Alfredo de Moraes, Samuel Santos Valenca, Manuella Lanzetti

**Affiliations:** 1 Graduate Program of Immunology and Inflammation, Universidade Federal do Rio de Janeiro, Rio de Janeiro 21941-590, Brazil; aline.cardoso357@gmail.com; 2 Instituto de Ciências Biomédicas, Universidade Federal do Rio de Janeiro, Rio de Janeiro 21941-590, Brazil; figueiredojunior.at@gmail.com (A.T.F.-J.); priscilla.mineiro@icb.ufrj.br (P.C.d.O.M.); evelynmott30@gmail.com (E.C.M.); carolinne28amorim@hotmail.com (C.S.d.A.); helfarma@yahoo.com.br (H.d.M.V.); casimiro.line@gmail.com (A.C.C.d.A.G.); joaomoraes@icb.ufrj.br (J.A.d.M.); manuellalanzetti@icb.ufrj.br (M.L.); 3 Instituto de Biofísica Carlos Chagas Filho, Universidade Federal do Rio de Janeiro, Rio de Janeiro 21941-902, Brazil; sabrinasodre@biof.ufrj.br (S.S.d.S.S.); pedroleme@biof.ufrj.br (P.L.S.); takiyacm@biof.ufrj.br (C.M.T.)

**Keywords:** chronic obstructive pulmonary disease (COPD), dimethyl fumarate (DMF), monomethyl fumarate (MMF), oxidative stress, inflammation

## Abstract

Chronic obstructive pulmonary disease (COPD) is characterized by chronic pulmonary inflammation and the destruction of the pulmonary parenchyma (emphysema), with only symptomatic treatment available. Molecules with antioxidant and anti-inflammatory properties, such as dimethyl fumarate (DMF), have shown therapeutic potential. This study evaluated the effects of DMF and its metabolite, monomethyl fumarate (MMF), on pulmonary inflammation induced by cigarette smoke (in vitro) and porcine pancreatic elastase (PPE) in mice (in vivo). In vitro, human pulmonary epithelial cells (PC-9) were treated with MMF at concentrations of 10, 30, and 100 µM and exposed to cigarette smoke extract (CSE) to assess cell viability, oxidative stress (ROS), lipid peroxidation, and nitrite production. In vivo, C57BL/6 mice were treated with DMF (30 and 100 mg/kg) during and after the induction of emphysema by PPE. ROS levels, total cell count in bronchoalveolar lavage fluid (BALF), lung histology, and the expression of oxidative stress proteins (SOD1 and HO-1) were analyzed. MMF reduced oxidative stress and lipid peroxidation under in vitro conditions. In vivo, DMF reduced ROS levels, inflammation, and prevented lung damage, such as alveolar enlargement. The expression of SOD1 and HO-1 was modulated by DMF treatment. The results suggest that DMF could be an effective therapeutic alternative for COPD, reducing oxidative stress and inflammation.

## 1. Introduction

Chronic obstructive pulmonary disease (COPD) is one of the leading respiratory diseases, with significant socioeconomic impact, affecting approximately 210 million people worldwide [1]. COPD is characterized by progressive airflow limitation and chronic inflammatory responses in the lungs, leading to structural alterations such as the destruction of lung parenchyma (emphysema) and loss of elasticity, resulting in irreversible airway obstruction [2]. The inflammation associated with COPD is mediated by macrophages, which release pro-inflammatory mediators and recruit other inflammatory cells, such as neutrophils and lymphocytes. These cells contribute to tissue damage and the exacerbation of the inflammatory response, triggering the release of reactive oxygen species (ROS) and nitrogen species (RNS) [3]. The imbalance between proteases and antiproteases, oxidative stress, and improper tissue repair are critical factors in the development of COPD [4]. Cigarette smoking is the primary etiological factor of COPD, and is associated with the release of proteolytic enzymes, such as elastase, which degrade elastin, a structural component of lung tissue [5]. Moreover, the experimental induction of emphysema is also performed by the intranasal instillation of porcine pancreatic elastase (PPE), which causes damage similar to that induced by cigarette smoke [6].

Currently, there is no cure for COPD, and treatment is symptomatic, consisting of bronchodilators, corticosteroids, and pulmonary rehabilitation. However, these treatments do not prevent disease progression [7]. Antioxidants have gained prominence as therapeutic options due to their potential to modulate oxidative stress and inflammation [8,9]. Studies have shown that dimethyl fumarate (DMF), an ester of fumaric acid, possesses anti-inflammatory and antioxidant properties [10,11,12]. DMF is already used in the treatment of psoriasis and multiple sclerosis, diseases in which oxidative stress and inflammation are exacerbated [13]. DMF acts as an antioxidant by activating the Nrf2/Keap1 pathway, which regulates the expression of essential antioxidant enzymes, such as glutathione S-transferase A2 (GSTA 2), heme oxygenase 1 (HO-1), and quinone oxidoreductase 1 (NQO1), promoting cellular detoxification [12]. Furthermore, DMF inhibits the translocation of NF-κB to the nucleus, attenuating inflammation [14]. The activation of Nrf2 by DMF has been associated with reduced inflammation in the airways and protection against oxidative stress [15], making it a potential therapeutic agent for inflammatory pulmonary diseases, including COPD.

Previous studies, such as that by Muralidharan and co-workers explored the use of DMF as an inhalable powder, leveraging its capacity to activate Nrf2. They demonstrated that breathable microparticles could reach the lower pulmonary tract, treating inflammation associated with COPD [10]. Given the limited efficacy of current treatments, there is an urgent need for new therapeutic approaches capable of modulating both oxidative stress and inflammation. Fumaric esters, such as DMF and its active metabolite monomethyl fumarate (MMF), show considerable therapeutic potential due to their antioxidant and anti-inflammatory properties. However, their specific impact on the treatment of COPD and the understanding of their mechanisms of action in this context still require further investigation. Thus, our study aims to evaluate the ability of DMF and MMF to modulate pulmonary inflammation and emphysema induced experimentally, using both in vivo (PPE) and in vitro (CSE) models.

## 2. Materials and Methods

### 2.1. Cell Culture and Cigarette Smoke Extract (CSE) Preparation

All reagents used in this study and described below were purchased from Merck KGaA (Darmstadt, Hesse, Germany), except where a different company is specified. The human lung epithelial cell line PC-9 was cultured in RPMI-1640 medium containing 50 U/mL penicillin and 100 µg/mL streptomycin and incubated at 37 °C in a 5% CO_2_ atmosphere. The preparation of CSE was performed as follows: smoke from one cigarette was bubbled into a 50 mL Falcon tube containing 10 mL of 1% fetal bovine serum (FBS) medium, and the pH was adjusted to 7.0. Under the flow, the CSE was filtered through a 0.22 µm filter, and the working dilution was prepared (1.25%, 2.5%, 5%, and 10%). After preparing the working dilution, the absorbance at 320 nm of the 100% CSE was measured and recorded.

### 2.2. MTT Assay

PC-9 cells were seeded in 96-well plates (Corning, Glendale, AZ, USA) at 3 × 10^3^ cells/well in RPMI-1640 with 10% FBS and incubated at 37 °C with 5% CO_2_ for 24 h. After adhesion, cells were treated with CSE (0.625–10%) and/or MMF (10, 30, 100 µM) for 1 or 24 h. Subsequently, 100 µL of MTT (5 mg/mL in PBS) in RPMI-1640 was added, and cells incubated for 4 h at 37 °C to form formazan crystals. The medium was removed, and crystals dissolved in 100 µL isopropyl alcohol with gentle agitation. Absorbance at 570 nm was measured using a microplate reader (PerkinElmer multilabel reader, Springfield, IL, USA). Cell viability was expressed as a percentage relative to untreated controls.

### 2.3. Lactate Dehydrogenase Activity Assay

PC-9 cells (9 × 10^4^ cells/well) were seeded in 24-well plates in RPMI-1640 with 10% FBS. After 24 h, cells were treated with MMF (10, 30, 100 µM) or CSE (0.625–10%) for 24 h. Cell viability was assessed by lactate dehydrogenase (LDH) activity using a commercial kit (Bioclin, Belo Horizonte, Brazil) following the manufacturer’s instructions. The assay measures LDH-catalyzed conversion of pyruvate to lactate in the presence of NADH, monitored as the decrease in absorbance at 340 nm. For the reaction, 4 µL of supernatant was added to 196 µL of working reagent in 96-well plates (triplicates). Absorbance was recorded every 30 s for 3 min using a microplate reader (PerkinElmer multilabel reader, Springfield, IL, USA). LDH activity was expressed as ΔA/min normalized to control.

### 2.4. NF-κB and Nrf2/ARE Luciferase Activity Assay

PC-9 cells (9 × 10^4^ cells/well) were seeded in 24-well plates in RPMI 1640 with 10% FBS. After 24 h, cells were transfected with an Nrf2- or NF-κB-responsive luciferase reporter plasmid (BPS Bioscience, San Diego, CA, USA) using Lipofectamine 2000 (Thermo Fisher Scientific, Waltham, MA, USA) in Opti-MEM containing 1% FBS, following the manufacturer’s protocol. After stimulation, cells and culture medium were transferred to white 96-well plates, and luciferase activity was assessed using the Dual Luciferase Assay System (BPS Bioscience, San Diego, CA, USA) on a Varioskan LUX reader (Thermo Fisher Scientific, Waltham, MA, USA).

### 2.5. Animals and Experimental Design

**Figure 1:**
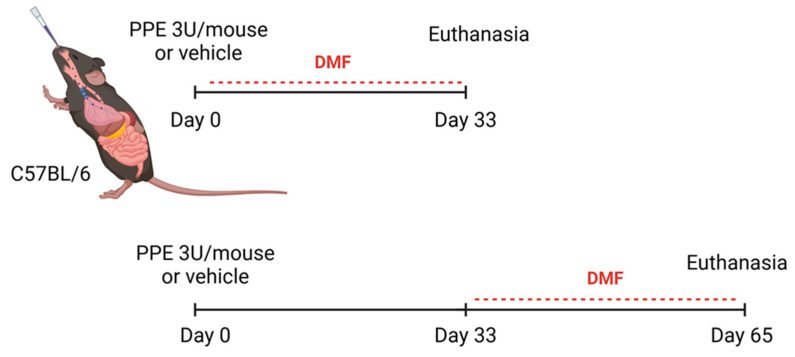
Schematic representation of the experimental design for the emphysema model in C57BL/6 mice. Animals were administered porcine pancreatic elastase (PPE, 3 U/mouse) or vehicle on Day 0. In the first experimental group (top), dimethyl fumarate (DMF) treatment was initiated on Day 0 and continued until Day 33, followed by euthanasia (study 1). Mice were divided into the following groups: control (instillation of the PPE vehicle [0.9% saline] and oral gavage of the DMF vehicle [0.5% carboxymethylcellulose in PBS]); DMF100 (instillation of the PPE vehicle and treatment control with DMF at 100 mg/kg); PPE (emphysema induced with PPE [3 U/mouse]); PPE + DMF30 (emphysema induced and treated concomitantly with DMF 30 mg/kg); PPE + DMF100 (emphysema induced and treated concomitantly with DMF 100 mg/kg). In the second experimental group (bottom), DMF treatment was initiated on Day 33 and maintained until Day 65, at which point euthanasia was performed (study 2). Mice were divided into the following groups: control (instillation of the PPE vehicle [0.9% saline] and oral gavage of the DMF vehicle [0.5% carboxymethylcellulose in PBS]); PPEr (emphysema induced with PPE and recovery period); PPEr + DMF100 (emphysema induced with PPE, followed by post-treatment with DMF 100 mg/kg for 32 days). In all cases, DMF was administered orally at 30 or 100 mg/kg, 3×/week. The solid black line represents the emphysema-PPE induction period (Day 0 to Day 33) and the recovery period (Day 34 to Day 65), while the dotted red line shows the period of DMF treatment administration.

This study was conducted at the Health Sciences Center (CCS) of the Federal University of Rio de Janeiro (UFRJ) and was previously approved by the UFRJ Ethics Committee under protocol number 016/22. C57BL/6 mice, 8 weeks of age, were used in the experiments. The animals were housed in ventilated racks in the Institute of Biomedical Sciences, maintained under a 12 h reversed light–dark cycle, with a controlled temperature of 21 ± 2 °C and relative humidity of 50 ± 10%, with free access to water and food. In the first experimental design, emphysema was induced by intranasal instillation of PPE at a dose of 3 U (units/mouse), diluted in sterile saline solution. DMF was diluted in a 0.5% carboxymethylcellulose vehicle and administered by oral gavage three times a week at doses of 30 and 100 mg/kg body weight (Figure 1). The animals were divided into the following groups: control (instillation of the PPE vehicle [0.9% saline] and oral gavage of the DMF vehicle [0.5% carboxymethylcellulose in PBS]); DMF100 (instillation of the PPE vehicle and treatment control with DMF at 100 mg/kg); PPE (emphysema induced with PPE [3 U/mouse]); PPE + DMF30 (emphysema induced and treated concomitantly with DMF 30 mg/kg); PPE + DMF100 (emphysema induced and treated concomitantly with DMF 100 mg/kg). Euthanasia of the animals occurred 33 days after the induction of emphysema (Figure 1). For the second experimental design, was included a recovery period after the induction of emphysema, with groups divided as follows: control (instillation of the PPE vehicle [0.9% saline] and oral gavage of the DMF vehicle [0.5% carboxymethylcellulose in PBS]); PPEr (emphysema induced with PPE and recovery period); PPEr + DMF100 (emphysema induced with PPE, followed by post-treatment with DMF 100 mg/kg for 32 days). Treatment started on day 33, after the recovery period, and animals were sacrificed on day 65 (Figure 1).

### 2.6. Western Blotting

Lung tissues were homogenized in RIPA buffer with protease inhibitors on ice, centrifuged at 12,000× *g* for 10 min at 4 °C, and supernatants collected as homogenates. Proteins (50 µg) from lung homogenates were separated by SDS-PAGE (8–12%) and transferred to nitrocellulose membranes. After blocking with 5% BSA in TBS-T (0.1% Tween-20), membranes were incubated for 2 h with primary antibodies: anti-SOD1 and anti-HO-1 (goat), anti-nitrotyrosine (PNK, rabbit) (Santa Cruz Biotechnology, Dallas, TX, USA), and anti-MMP-9 (rabbit, Invitrogen, Thermo Fisher Scientific, Waltham, MA, USA). After washing, membranes were incubated with HRP-conjugated anti-goat or anti-rabbit secondary antibodies (Santa Cruz Biotechnology, Dallas, TX, USA) and developed using chemiluminescence reagents (Thermo Fisher Scientific, Waltham, MA, USA) on a ChemiDoc MP system (Bio-Rad Laboratories, Hercules, CA, USA). Membranes were reprobed for β-actin (Merck KGaA, Darmstadt, Germany). In some cases, multiple proteins were evaluated from the same blot, and the loading control from that blot was then used for more than one protein. Band intensity was quantified using ImageJ software v. 1.54p (NIH, Bethesda, MD, USA) and expressed as arbitrary units.

### 2.7. Nitrite Assay

Nitrite levels were determined using the Griess reaction. Briefly, 50 µL of lung homogenate was diluted with 50 µL of distilled water, transferred in duplicate to a 96-well plate, and mixed with 50 µL of 1% sulfanilamide in 2.5% phosphoric acid. After 10 min at room temperature, 50 µL of 0.1% naphthylethylenediamine in 2.5% phosphoric acid was added. A standard curve was prepared with nitrite concentrations ranging from 0 to 100 µM. Absorbance was measured at 540 nm using a microplate reader (PerkinElmer multilabel reader, Springfield, IL, USA). Results were expressed as µM nitrite per mg of protein.

### 2.8. Thiobarbituric Acid Reactive Substances (TBARS)

Lipid peroxidation was assessed by measuring malondialdehyde (MDA) using the thiobarbituric acid reactive substances (TBARS) assay. Briefly, 150 µL of lung homogenate was mixed with 150 µL of 10% trichloroacetic acid, centrifuged at 3600× *g* for 15 min, and 150 µL of the supernatant was combined with 150 µL of 0.67% thiobarbituric acid. The mixture was heated at 95 °C for 10 min. A standard curve was prepared using 1,1,3,3-tetramethoxypropane (0.625–100 µM). Absorbance was measured at 532 nm using a microplate reader (PerkinElmer multilabel reader, Springfield, IL, USA). Results were expressed as µM MDA per mg protein.

### 2.9. Histology and Morphometry

The chest was opened via a ventral incision, and the right ventricle perfused with saline. The left lung was fixed in 10% neutral buffered formalin (pH 7.2) for 48 h, processed through graded ethanol (70–100%) for 60 min each, cleared in three xylene baths, and embedded in paraffin at 60 °C. Tissues were then immersed in liquid paraffin at 55 °C to remove residual xylene and cooled for solidification. Sections (5 µm) were cut with a microtome, mounted on slides, and stained with hematoxylin–eosin (H&E) and orcein. Macrophages were counted in 15 random fields per slide, expressed as inflammatory cells/field. Slides were digitized using a Zeiss Primo Star microscope (ZeissVision, Oberkochen, Baden-Württemberg, Germany).

Pulmonary emphysema was quantified histologically based on the degree of increase in alveolar air spaces, measured by the mean linear intercept (Lm) and determined by counting 15 random fields per animal in H&E-stained sections. The method involves counting the number of times alveolar septa intersect a grid of parallel lines within a bronchiole-free field [16]. Lm was calculated using the formula:

Lm = L (tot)/L (i)(1)

where L (tot) is the total length of the grid lines, and L (i) is the number of intercepts by the alveolar septa. Orcein stained sections were subjected to stereology through the volume density (Vv) of the elastic fibers [17]. A test grid with 21 points within a defined area was used to avoid overestimating the structures. The volume density of the elastic fibers was calculated by dividing the number of points that intersect the elastic fibers (Pp) by the total number of points in the grid (Pt) using the formula:

Vv [a] = Pp/Pt(2)

### 2.10. Immunohistochemistry

Lung sections were blocked with 3% BSA, washed in PBS with 2.5% Triton X-100, and incubated with 10% hydrogen peroxide in PBS. After washing, primary antibodies against phosphorylated Nrf2 (pNrf2), Keap1, and phosphorylated NF-κB (pNF-κB) (Invitrogen, Thermo Fisher Scientific, Waltham, MA, USA) were applied, followed by detection using a dextran polymer system (EnVision kit, Dako, Carpinteria, CA, USA) and DAB chromogen. Sections were counterstained with hematoxylin, and images captured using a Zeiss Primo Star microscope (ZeissVision, Oberkochen, Baden-Württemberg, Germany). Positive staining for pNrf2, Keap1, and pNF-κB was quantified by automated image analysis (ImageJ, NIH, Bethesda, MD, USA) in 10 random fields from 3 lung samples per group.

### 2.11. Detection of Reactive Oxygen Species In Vitro and In Vivo

After euthanasia, tracheal cannulation was performed, and 1.5 mL of saline was instilled into the lungs (3 × 500 µL) to collect BALF. Samples were centrifuged at 600× *g* for 10 min at 4 °C (Eppendorf Vertrieb Deutschland GmbH, Wesseling-Berzdorf, Germany); the supernatant was stored at −20 °C, and the cell pellet immediately used for ROS analysis. BALF cells or PC-9 cultures were resuspended in phenol-red–free HBSS and seeded at 4 × 10^4^ cells/well in black-walled, clear-bottom 96-well plates (Corning, Glendale, AZ, USA). Intracellular ROS and peroxynitrite were detected using 2′,7′-dichlorodihydrofluorescein diacetate (H_2_DCFDA) and aminophenyl fluorescein (APF) probes (Thermo Fisher Scientific, Waltham, MA, USA) at 5 µM in HBSS, followed by 1 h incubation at 37 °C with 5% CO_2_. After washing, fluorescence (excitation 495 nm/emission 525 nm) was measured in a microplate reader (PerkinElmer, Springfield, IL, USA).

### 2.12. Statistical Analysis

The values were expressed as mean ± standard deviation (SD), and differences between groups were evaluated using a one-way ANOVA test followed by Bonferroni post hoc test. Statistical analyses were performed using GraphPad Prism software (version 9.0, San Diego, CA, USA). Statistical significance was defined as a *p*-Value < 0.05.

## 3. Results

### 3.1. Effects of CSE and Monomethyl Fumarate (MMF) on Cell Viability and Oxidative Stress

**Figure 2:**
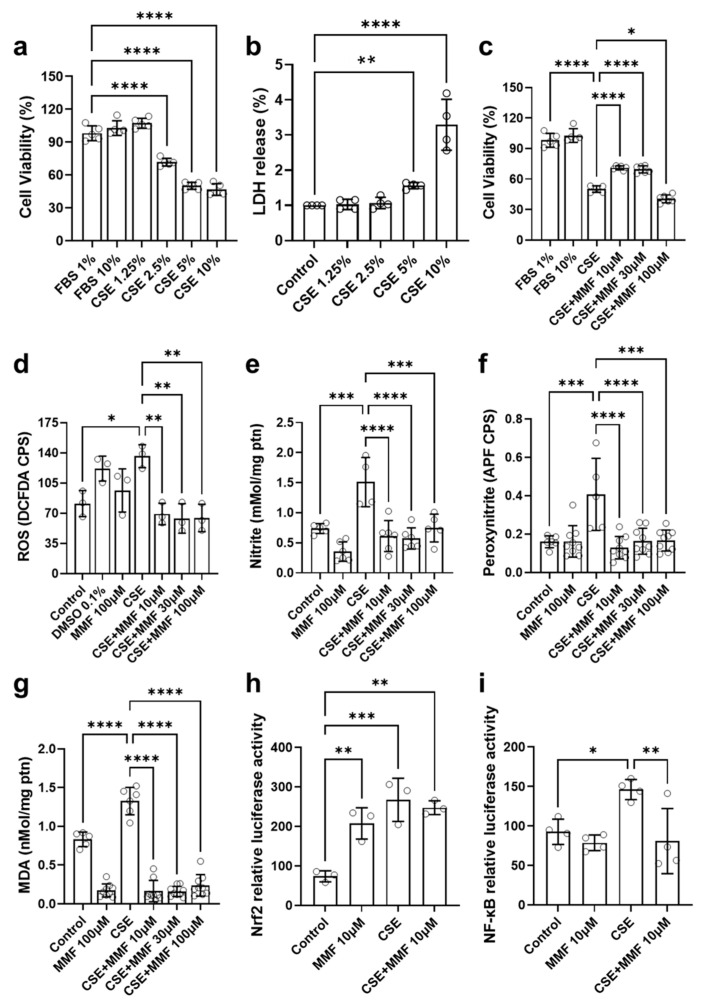
Effects of cigarette smoke extract (CSE) and dimethyl fumarate (DMF) on cell viability, lactate dehydrogenase (LDH) release, reactive oxygen species (ROS) production, nitrite and peroxynitrite levels, malondialdehyde (MDA) concentration, Nrf2-dependent luciferase activity, and NF-κB-dependent luciferase activity in lung (PC-9) cells. (**a**) Cell viability of lung cells exposed to different concentrations of CSE (1.25%, 2.5%, 5%, and 10%); (**b**) LDH release in lung cells exposed to different concentrations of CSE; (**c**) Cell viability of lung cells treated with DMF (10 µM, 30 µM, and 100 µM) in the presence of 10% CSE; (**d**) ROS levels in lung cells treated with DMF in the presence of CSE; (**e**) Nitrite concentration in lung cells treated with DMF in the presence of CSE; (**f**) Peroxynitrite levels in lung cells treated with DMF in the presence of CSE; (**g**) MDA concentration in lung cells treated with DMF in the presence of CSE; (**h**) Nrf2-dependent luciferase activity in lung cells treated with DMF in the presence of CSE; (**i**) NF-κB-dependent luciferase activity in lung cells treated with DMF in the presence of CSE. Data are expressed as mean ± standard deviation from independent experiments. Statistical analysis was performed by using one-way ANOVA with Bonferroni post hoc test. * *p* < 0.05; ** *p* < 0.01; *** *p* < 0.001; **** *p* < 0.0001. The sample size for each column in the graphs is indicated by open circles.

PC-9 cells were incubated with varying concentrations of CSE and MMF. It was observed that, after 1 h of incubation, neither CSE nor MMF altered cell viability, as indicated by the MTT assay (Figure S1a–c). However, after 24 h, CSE significantly reduced cell viability across all tested concentrations (Figure 2a), except for the 1.25% concentration. The LDH assay was employed to confirm the toxicity of CSE on the cells, revealing an increase in toxicity levels at concentrations of 5% and 10% (Figure 2b). In contrast, MMF exhibited no toxicity at the tested concentrations, as demonstrated by both the MTT and LDH assays (Figure S1d,e). Furthermore, at concentrations of 10 µM and 30 µM, MMF partially prevented the reduction in cell viability induced by CSE exposure, suggesting a potential protective effect against cytotoxicity (Figure 2c).

Levels of ROS, measured using the fluorescent 2′,7′-dichlorofluorescein (DCF) signal, were comparable to the control in the MMF-treated group, whereas they were elevated in the CSE-treated group. MMF, at all tested concentrations, prevented the increase in ROS (Figure 2d). A similar pattern was observed in nitrite quantification using the Griess method (Figure 2e), where only the CSE group exhibited elevated nitrite concentrations compared to the control group, while all tested MMF concentrations prevented this increase. Figure 2f shows peroxynitrite quantification using the fluorescent probe APF, indicating a significant increase in the CSE group, which was mitigated in the MMF-treated groups across all concentrations. These findings align with the results of the lipid peroxidation assay, assessed via the TBARS method, which indirectly measures oxidative damage (Figure 2g). The CSE group exhibited higher MDA concentrations compared to the control group, while all tested MMF concentrations prevented this oxidative damage.

The luciferase assay was conducted to measure the activation of Nrf2 and NF-κB. It was observed that the MMF group exhibited increased Nrf2 activation (Figure 2h), consistent with the expected mechanism of action of this compound. CSE also induced an increase in Nrf2 activation, and MMF treatment did not alter this CSE-induced Nrf2 activation. However, in the assay measuring NF-κB activation, CSE increased the signal, whereas MMF treatment prevented this change (Figure 2i). For these assays, an MMF concentration of 10 µM was used, as all concentrations tested in prior assays effectively reduced oxidative parameters.

### 3.2. Effects of DMF on the Elastase-Induced Pulmonary Emphysema (Study 1)

**Figure 3:**
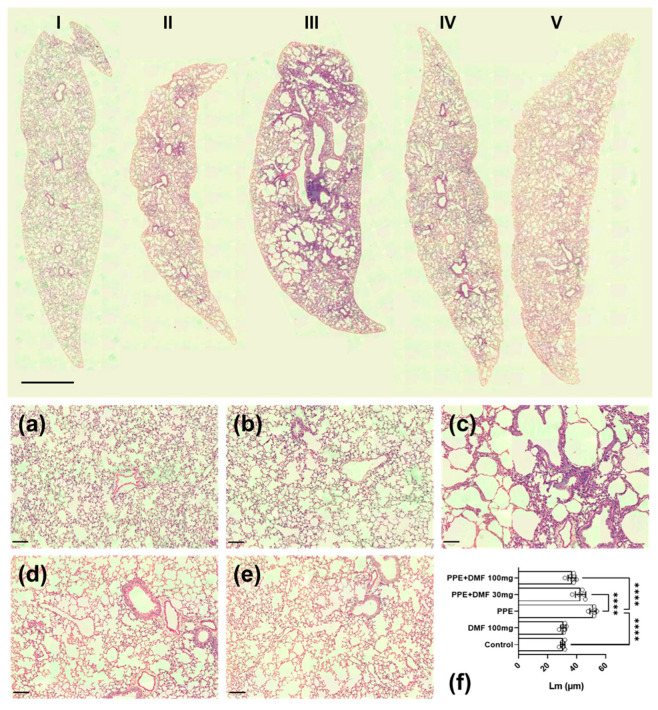
Histological sections of lungs stained with Hematoxylin and Eosin (H&E). On top, slides were scanned and represent each experimental group as indicated. The control group received only the vehicle (**I**); the DMF group received dimethyl fumarate (DMF) at 100 mg/kg (**II**); the PPE group was instilled with elastase, showing signs of emphysema and alveolar destruction (**III**); the PPE + DMF30 group was instilled with elastase and concomitantly treated with DMF at 30 mg/kg (**IV**); and the PPE + DMF100 group was instilled with elastase and concomitantly treated with DMF at 100 mg/kg (**V**). The latter two groups exhibited significant improvement in lung histology compared to the PPE group. Objective: 2×. Scale bar = 2 mm. On down, representative images of lung histology showing that DMF prevented the development of porcine pancreatic elastase (PPE)-induced pulmonary emphysema in a murine experimental model. (**a**) Control group; (**b**) DMF group (100 mg/kg); (**c**) PPE group (3 U/mouse); (**d**) PPE group treated with DMF 30 mg/kg; (**e**) PPE group treated with DMF 100 mg/kg; (**f**) Quantification of mean alveolar diameter (Lm). Objective: 20×, scale bar = 100 µm. Data are expressed as mean ± standard deviation from independent experiments. Statistical analysis was performed by using one-way ANOVA with Bonferroni post hoc test. **** *p* < 0.0001. The sample size for each column in the graphs is indicated by open circles.

Figure 3 presents a representative histological section at low magnification (panoramic view) of a mouse lung from each group. I is control, II is DMF 100 mg/kg, III is PPE, IV is PPE + DMF 30 mg/kg and V is PPE + DMF 100 mg/kg. Tissue differences are evident, particularly due to the presence of emphysematous lesions in the PPE group (III). Higher-magnification representative images are shown in the same panel. The control group exhibited intact and preserved alveolar structures with appropriate diameters (Figure 3a). The DMF group showed no significant morphological changes in pulmonary histoarchitecture, resembling the control group (Figure 3b). The PPE group displayed morphological changes characteristic of pulmonary emphysema, including an increased mean alveolar diameter, as evidenced by histological analysis (Figure 3c). Figure 3d,e depict groups with PPE-induced emphysema treated concomitantly with DMF at doses of 30 mg/kg and 100 mg/kg, respectively. In both groups, the development of emphysema was inhibited, with superior outcomes observed at the higher dose. The graph in Figure 3f quantifies the mean linear intercept from histological sections, confirming the macro- and microscopic observations.

**Figure 4:**
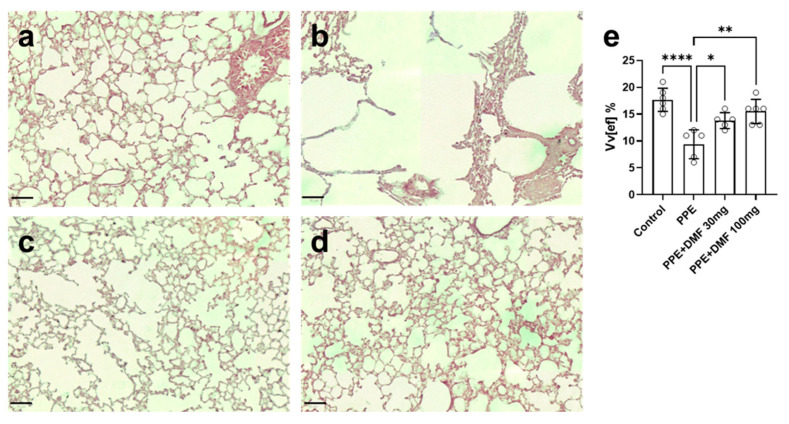
Representative images of lung elastic fibers stained with orcein. Dimethyl fumarate (DMF) preserved elastic fibers during porcine pancreatic elastase (PPE)-induced pulmonary emphysema in a murine experimental model. (**a**) Control group. (**b**) PPE group (3 U/mouse). (**c**) PPE group treated with DMF 30 mg/kg. (**d**) PPE group treated with DMF 100 mg/kg. (**e**) Stereological analysis of elastic fibers, quantifying the volume density of the pulmonary elastic component (Vv elastic fibers). Objective: 40×, scale bar = 50 µm. Data are expressed as mean ± standard deviation from independent experiments. Statistical analysis was performed by using one-way ANOVA with Bonferroni post hoc test. * *p* < 0.05; ** *p* < 0.01; **** *p* < 0.0001. The sample size for each column in the graphs is indicated by open circles.

These morphological changes were accompanied by a reduction in pulmonary elastic fiber distributions, as evidenced by orcein staining (Figure 4). Figure 4a represents the control group, showing characteristic staining of preserved elastic fibers in the alveolar septa. In the emphysematous group (Figure 4b), reduced orcein staining indicated a decrease in elastic fiber tissue distribution. Figure 4c,d demonstrate that elastic fibers were preserved in the DMF-treated groups at both 30 mg/kg and 100 mg/kg doses. Stereological analysis of elastic fibers, quantifying the volume density of the pulmonary elastic component, revealed that DMF treatment prevented the PPE-induced reduction in these tissue structures, with the 100 mg/kg dose being the most effective (Figure 4e).

Mononuclear cells (indicated by arrows) in the pulmonary alveoli were quantified in Giemsa-stained slides (Figure S2). The DMF-treated control group (Figure S2b) exhibited a slightly higher number of mononuclear cells compared to the vehicle-only control group (Figure S2a). Additionally, a significant increase in mononuclear cell numbers was observed in the PPE group (Figure S2c) relative to the control group. DMF treatment at doses of 30 mg/kg and 100 mg/kg prevented the increase in mononuclear cell numbers in the lungs of PPE-instilled mice (Figure S2d,e). The morphometry (counting) of cells/field is confirmed in Figure S2f.

### 3.3. Effects of DMF on Oxidative Stress During Elastase-Induced Pulmonary Emphysema (Study 1)

**Figure 5:**
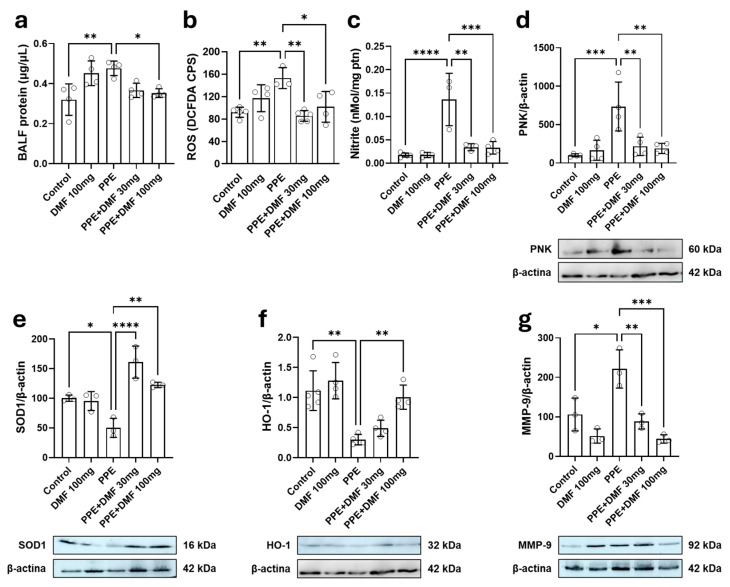
Effects of dimethyl fumarate (DMF) on bronchoalveolar lavage fluid (BALF) protein content, reactive oxygen species (ROS), nitrite, nitrotyrosine (product of tyrosine nitration mediated by reactive nitrogen species, PNK), superoxide dismutase 1 (SOD1), heme oxygenase 1 (HO-1), and matrix metalloproteinase 9 (MMP-9) in a murine model of porcine pancreatic elastase (PPE)-induced pulmonary emphysema. (**a**) BALF protein concentration; (**b**) ROS levels in BALF cells, measured by DCFDA; (**c**) Nitrite concentration in lung tissue; (**d**) PNK expression in lung tissue, normalized to β-actin, with Western blot images below; (**e**) SOD1 expression, normalized to β-actin; (**f**) HO-1 expression, normalized to β-actin; (**g**) MMP-9 expression, normalized to β-actin. In some cases (HO-1 and PNK), multiple proteins were evaluated from the same blot, and the loading control from that blot was then used for more than one protein. Data represent mean ± standard deviation from independent experiments. Statistical analysis was performed using one-way ANOVA with Bonferroni post hoc test (* *p* < 0.05; ** *p* < 0.01; *** *p* < 0.001; **** *p* < 0.0001). Sample sizes for each group are indicated by open circles. Uncropped Western blots for PNK, SOD1, HO-1, and MMP-9 are provided in Supplementary Figures S4–S7.

First, we measured tissue and cellular damage by total protein content in BALF, reflecting the loss of capillary integrity (and the blood-air barrier) with plasma content leakage into the alveoli. The PPE group exhibited a significant increase in BALF protein content compared to the control group (Figure 5a). Only the 100 mg/kg DMF dose prevented this increase during PPE-induced emphysema development. Then, markers of oxidative stress were investigated in the lung and BALF. ROS levels in BALF cells were elevated in the PPE group compared to the control group (Figure 5b). However, both DMF doses (30 mg/kg and 100 mg/kg) prevented the increase in ROS during PPE-induced emphysema development. Nitrite levels in lung homogenates were also elevated in the PPE group compared to the control group (Figure 5c). In the PPE + DMF30 and PPE + DMF100 groups, a significant reduction in nitrite levels was observed compared to the PPE group. Elevated nitrite levels may contribute to nitrosative stress, prompting the evaluation of PNK expression by Western blot as an indicator of protein nitration. An increase in PNK was observed in the PPE group compared to the control group, which DMF at both 30 mg/kg and 100 mg/kg doses prevented, even during PPE-induced emphysema development (Figure 5d). The antioxidant enzymes SOD1 (Figure 5e) and HO-1 (Figure 5f) were analyzed by Western blot as well. A reduction in SOD1 and HO-1 expression was observed in the PPE group compared to the control group. DMF treatment at both 30 mg/kg and 100 mg/kg doses prevented the reduction in SOD1 expression during PPE-induced emphysema development. However, for HO-1, only the 100 mg/kg DMF dose prevented the reduction in this enzyme in PPE-treated mice. Lastly, the expression of MMP-9, a metalloproteinase responsible for extracellular matrix components and produced by macrophages, neutrophils, and epithelial cells, was analyzed. An increase in MMP-9 expression was observed in PPE-treated mice compared to the control group (Figure 5g). Mice treated with DMF at both 30 mg/kg and 100 mg/kg doses did not exhibit this pattern, even following PPE instillation.

**Figure 6:**
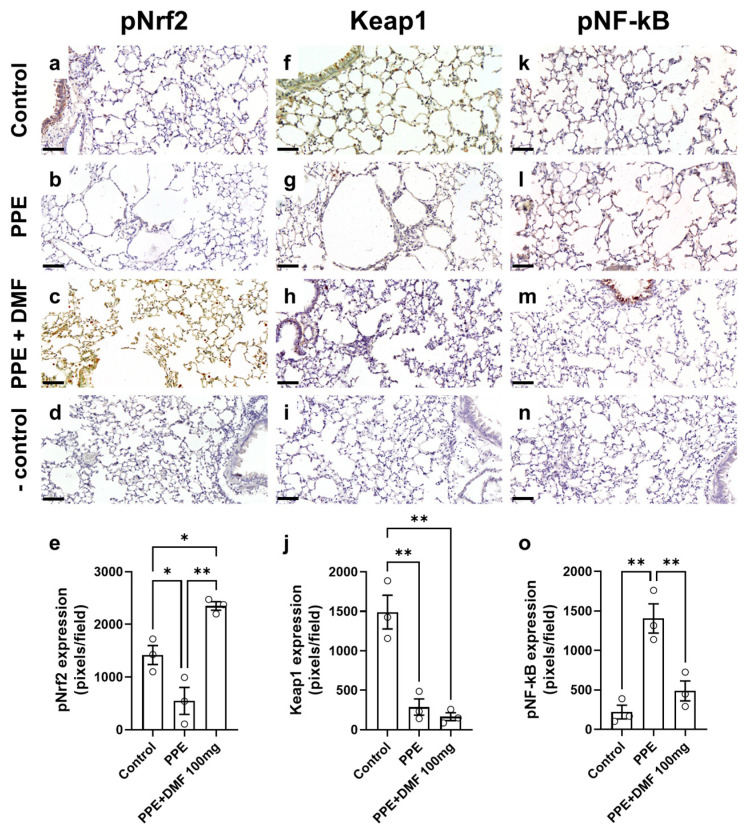
Representative imagens of lung immunohistochemistry for phosphorylated Nrf2, NF-κB, and Keap1 during porcine pancreatic elastase (PPE)-induced pulmonary emphysema in a murine experimental model. Diaminobenzidine was employed as a chromogen in the final step of the reaction. pNrf2, Keap1, and pNF-κB were visualized as brown staining in a subset of cells. pNrf2: (**a**) control group; (**b**) PPE group; (**c**) PPE + DMF (100 mg/kg) group; (**d**) negative control (non-primary antibody is used); (**e**) quantification of pNrf2 tissue expression. Keap1: (**f**) control group; (**g**) PPE group; (**h**) PPE + DMF (100 mg/kg) group; (**i**) negative control (non-primary antibody is used); (**j**) quantification of Keap1 tissue expression. pNF-κB: (**k**) control group; (**l**) PPE group; (**m**) PPE + DMF (100 mg/kg) group; (**n**) negative control (non-primary antibody is used); (**o**) quantification of Keap1 tissue expression. Data are expressed as mean ± standard deviation from independent experiments. Statistical analysis was performed by using one-way ANOVA with Bonferroni post hoc test. * *p* < 0.05 and ** *p* < 0.01. The sample size for each column in the graphs is indicated by open circles. Objective: 20×, scale bar = 100 µm.

To evaluate the activation profile of Nrf2 in our emphysema model, we performed immunohistochemistry for pNrf2 in mouse lung tissue. The control group (Figure 6a) exhibited minimal pNrf2 expression. In contrast, the group that received PPE and was treated with the vehicle (Figure 6b) showed reduced pNrf2 tissue expression compared to the control group. In the group treated with 100 mg/kg DMF and instilled with PPE (Figure 6c), pNrf2 tissue expression was effectively restored relative to the PPE group and was notably elevated compared to the control group (Figure 6e). A negative control (without primary antibody) is shown in Figure 6d.

We also performed immunohistochemistry for Keap1. Strong Keap1 expression was observed in the control group (Figure 6f), whereas both the PPE group (Figure 6g) and the PPE + DMF 100 mg/kg group (Figure 6h) exhibited reduced Keap1 tissue expression compared to the control group. A negative control (without primary antibody) is shown in Figure 6i. Quantification of positive Keap1 staining corroborated the visual analysis (Figure 6j).

Finally, immunohistochemistry was performed to evaluate the expression of pNF-κB in mouse lung tissue. The control group exhibited low pNF-κB tissue expression (Figure 6k). In comparison, the PPE group showed increased pNF-κB tissue expression (Figure 6l) relative to the control, whereas the PPE + DMF 100 mg group (Figure 6m) demonstrated a reduction in the expression of this transcription factor. Quantification of pNF-κB tissue expression is provided in Figure 6o, with the negative control shown in Figure 6n.

### 3.4. DMF Treatment Reverses Elastase-Induced Pulmonary Emphysema (Study 2)

**Figure 7:**
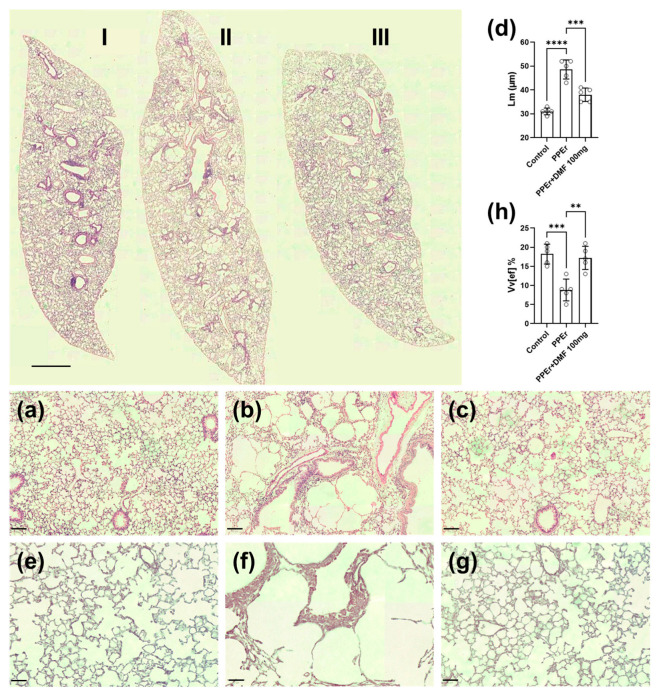
Histological sections of lungs stained with Hematoxylin and Eosin (H&E) and Orcein. On top, slides were scanned and represent each experimental group as indicated. The control group received only the vehicle (**I**); the PPEr group was instilled with elastase and maintained until 65 days, showing few signs of emphysema and alveolar destruction (**II**); the PPEr + DMF100 group was instilled with elastase and treated with DMF at 100 mg/kg started at day 33 (**III**). The latter group exhibited significant improvement in lung histology compared to the PPEr group. Objective: 2×. Scale bar = 2 mm. On down, representative images of lung histology stained with H&E (**a**–**c**) or orcein (**e**–**g**) showing that DMF reverted emphysema lesions caused by porcine pancreatic elastase (PPE) in a murine experimental model. (**a** or **e**) Control group; (**b** or **f**) PPEr group (3 U/mouse); (**c** or **g**) PPE group treated with DMF 100 mg/kg; (**d**) Quantification of mean alveolar diameter (Lm); (**h**) Volume density of elastic fibers by stereology. Objective: 20× for H&E with scale bar = 100 µm. Objective: 40× for orcein with scale bar = 50 µm. Data are expressed as mean ± standard deviation from independent experiments. Statistical analysis was performed by using one-way ANOVA with Bonferroni post hoc test. ** *p* < 0.01; *** *p* < 0.001; **** *p* < 0.0001. The sample size for each column in the graphs is indicated by open circles.

In this second experimental model of PPE-induced pulmonary emphysema, three groups were evaluated: control (PPE vehicle via intranasal (i.n.) route and DMF vehicle via oral (p.o.) route); PPEr (emphysema induced, with 32 days for recovery from the pathological condition); and PPEr + DMF100 (emphysema induced and treated with 100 mg/kg DMF starting on day 33 post-elastase instillation) (See Figure 1 for experimental design overview). Figure 7 presents panoramic lung images from scanned slides. The control group exhibited preserved histological structures (Figure 7I), while the PPEr group still displayed areas of pulmonary emphysema (Figure 7II). The PPEr + DMF100 group showed a histological pattern consistent with pulmonary tissue repair compared to the PPEr group (Figure 7III).

Figure 7a shows a histological section of a control group mouse lung with intact and preserved alveolar structures of appropriate diameters. PPE induced morphological changes characteristic of pulmonary emphysema (Figure 7b), including an increased mean alveolar diameter, as observed in the histological analysis. Figure 7c represents the group with PPE-induced emphysema treated with 100 mg/kg DMF, demonstrating reversal of emphysema. Figure 7d quantifies the mean alveolar diameter of these groups in the respective histological sections, confirming the morphological observations.

These morphological changes were accompanied by a reduction in pulmonary elastic fiber, as evidenced by orcein staining (Figure 7e–g). Figure 7e represents the control group, showing characteristic staining of preserved elastic fibers in the alveolar septa. In the emphysematous group (Figure 7f), reduced orcein staining indicated a decrease in elastic fiber tissue distribution. DMF treatment at 100 mg/kg restored elastic fibers in the alveolar septa (Figure 7g). Stereological analysis of elastic fibers, quantifying the volume density of the pulmonary elastic component, revealed that DMF treatment restored these tissue structures following PPE-induced emphysema (Figure 7h).

The therapeutic effects of DMF were also evident in the reversal of mononuclear cell numbers (Figure S3). Figure S3a shows that the control group exhibited few mononuclear cells in the alveoli (arrows). The PPEr group (Figure S3b) displayed an increased number of mononuclear cells compared to the control group. DMF treatment at 100 mg/kg effectively reduced the number of mononuclear cells in the alveoli of elastase-instilled mice (Figure S3c). Figure S3d presents a graph of the morphometric quantification of mononuclear cells in the pulmonary alveoli from the analyzed slides, with statistical data corroborating the morphological observations.

### 3.5. DMF Treatment Reduces Oxidative Stress Following Emphysema (Study 2)

**Figure 8:**
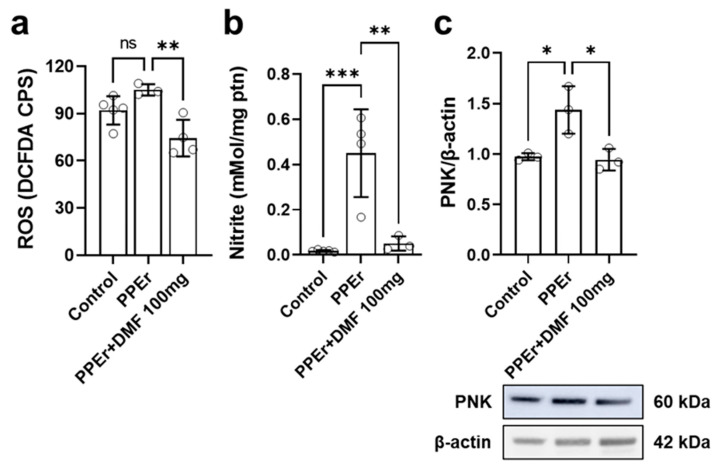
Effects of dimethyl fumarate (DMF) on the reactive oxygen species (ROS), nitrite levels, and nitrotyrosine (product of tyrosine nitration mediated by reactive nitrogen species) (PNK) during porcine pancreatic elastase (PPE)-induced pulmonary emphysema in a murine experimental model. (**a**) ROS levels in BALF cells, measured by DCF; (**b**) Nitrite concentration in lung tissue; (**c**) PNK expression in lung tissue, normalized to β-actin. The image below the graph shows Western blot results for PNK and β-actin. Data are expressed as mean ± standard deviation from independent experiments. Statistical analysis was performed by using one-way ANOVA with Bonferroni post hoc test. The "ns" in Figure 8a indicates no statistical significance. * *p* < 0.05; ** *p* < 0.01; *** *p* < 0.001. The sample size for each column in the graphs is indicated by open circles. Uncropped and unedited Western blot membrane for PNK is provided in supplementary material (Figure S8).

In the previous in vivo experiment, DMF was administered concomitantly with elastase-induced emphysema development. In this treatment phase, it was observed that the PPEr group showed only a trend toward increased ROS (*p* = 0.0571), which is understandable given the 64-day interval since the elastase stimulus (Figure 8a). However, the group treated with 100 mg/kg DMF exhibited a substantial reduction in ROS levels following pulmonary emphysema. Subsequently, a significant increase in nitrite levels was observed in the PPEr group compared to the control group (Figure 8b). The PPEr +DMF100 group showed reduced nitrite levels compared to the PPEr group, resembling the control group. Lastly, tissue PNK expression (Figure 8c), measured by Western blotting, was higher in the PPEr group compared to the control group. Again, DMF treatment at 100 mg/kg reduced tissue PNK expression compared to the PPEr group.

## 4. Discussion

The findings of this study underscore the therapeutic potential of MMF and DMF in managing conditions driven by oxidative stress and pulmonary inflammation. In the in vitro model, MMF exhibited a fully protective effect by mitigating the CSE-induced reduction in cell viability. This suggests that MMF may modulate cellular pathways linked to cell death, likely through regulation of the redox balance, as evidenced by decreased levels of ROS, nitrite, and peroxynitrite. Additionally, the reduction in lipid peroxidation, marked by lower MDA levels in MMF-treated groups, supports its antioxidant properties. These results align with prior studies showing that cigarette smoke elevates ROS production, triggering cellular damage and contributing to pulmonary emphysema [18,19,20,21,22]. Thus, MMF’s protective role may stem from its ability to stabilize redox pathways and preserve cellular metabolism.

The antioxidant capacity of MMF was further confirmed by its ability to prevent ROS accumulation in CSE-exposed cells. While CSE markedly increased ROS production, MMF treatment across all tested concentrations effectively blocked this rise, likely by activating intracellular antioxidant mechanisms. It is well-documented that fumarates activate the Nrf2/ARE pathway, upregulating antioxidant genes such as NQO1 and HO-1 [23,24,25]. This mechanism neutralizes ROS, prevents redox collapse, and reduces the cytotoxic impact of cigarette smoke, offering a plausible explanation for MMF’s efficacy. Moreover, MMF’s suppression of peroxynitrite and nitrite levels highlights its dual antioxidant and anti-inflammatory roles. Peroxynitrite, a highly reactive molecule formed from ROS and nitric oxide, causes severe oxidative damage to lipids, proteins, and DNA [26,27,28]. The observed reduction in these markers suggests that MMF may either inhibit nitric oxide synthase activity or neutralize ROS prior to peroxynitrite formation, consistent with previous reports of fumarates mitigating inflammation and oxidative damage in pulmonary disease models [29,30,31,32].

The decrease in MDA levels in MMF-treated groups further indicates protection against oxidative membrane damage. CSE significantly elevated MDA, reflecting lipid peroxidation induced by oxidative stress, whereas MMF prevented this increase, likely stabilizing cell membranes via antioxidant pathways. This is critical, as lipid peroxidation products like MDA can amplify inflammation and exacerbate cell injury [33]. These findings position MMF as a promising agent for protecting cells from CSE-induced damage and enhance our understanding of its antioxidant mechanisms.

In the in vivo elastase-induced emphysema model, DMF demonstrated robust protective effects, preserving alveolar morphology, elastic fiber density, and reducing alveolar macrophage infiltration. These outcomes are likely tied to its modulation of oxidative stress, as DMF-treated groups exhibited significant reductions in ROS, nitrite, and peroxynitrite levels. Furthermore, DMF restored the expression of antioxidant enzymes SOD1 and HO-1 while decreasing matrix metalloproteinase-9 (MMP-9), suggesting a role in regulating tissue remodeling. Histological analysis revealed that PPE induced substantial increases in mean alveolar diameter, indicative of emphysematous tissue destruction. DMF treatment, particularly at 100 mg/kg, markedly attenuated these changes, supporting its capacity to limit structural damage. Intriguingly, the tissue expression of pNrf2 was diminished in the emphysema group, consistent with prior investigations [6,18,34,35]. While Keap1 was reduced in vivo, in vitro assessment would clarify if MMF directly modulates Keap1 under CSE conditions. Literature indicates that DMF disrupts Keap1-Nrf2 interactions in lung cells exposed to oxidative stressors, supporting our observed Nrf2 activation [35]. Since Nrf2 serves as a critical defense mechanism against the development of emphysema, treatment with DMF effectively preserved pNrf2 activation, indicating that this may be one of the mechanisms through which DMF exerts its protective effects on murine pulmonary tissue. This is consistent with prior studies demonstrating the anti-inflammatory and antioxidant properties of fumaric acid esters in pulmonary models [29,30,31].

The preservation of elastic fiber density, assessed via orcein staining, further underscores DMF’s efficacy. PPE exposure reduced elastic fiber staining, reflecting matrix degradation central to emphysema progression. In contrast, DMF-treated groups, especially at 100 mg/kg, maintained fiber density near control levels, potentially by downregulating proteolytic enzymes like MMPs. The observed reduction in MMP-9 expression corroborates this hypothesis and suggests DMF regulates extracellular matrix remodeling [36]. Additionally, DMF reduced alveolar macrophage numbers, a key indicator of inflammation. PPE significantly increased macrophage infiltration, reflecting an exaggerated immune response, whereas DMF treatment attenuated this effect, possibly by suppressing NF-κB-mediated inflammatory pathways, as reported here by immunostaining and in prior studies [3,4,37]. Our results align with prior evidence that DMF activates Nrf2 to mitigate oxidative stress and inflammation in COPD models, such as through Nrf2/HO-1 signaling to suppress NLRP3-related pyroptosis in cigarette smoke-induced inflammation [15]. Additionally, microparticulate formulations of DMF have been shown to target pulmonary inflammation via Nrf2 activation [10]. Future studies using Nrf2-deficient models could further confirm this dependency. This dual action on oxidative stress and inflammation highlights DMF’s multifaceted protective role.

Notably, in a second in vivo model, post-treatment with DMF not only prevented but also partially reversed emphysema-related changes, including alveolar morphology recovery and normalization of oxidative stress markers (ROS, nitrite, and PNK). The Nrf2/ARE pathway likely underpins this effect, promoting antioxidant gene expression and mitigating chronic oxidative damage [38,39]. These findings position DMF as a promising candidate for treating established pulmonary conditions.

A key limitation of our study is the absence of Nrf2 knockout models, which would have allowed us to directly validate the pathway’s dependency. While our findings—based on luciferase assays and the modulation of downstream enzymes—strongly suggest Nrf2’s involvement, genetic validation would have provided definitive evidence of causality. This aligns with existing literature that highlights the crucial role of Nrf2 in emphysema models [6,35]. Furthermore, our study did not evaluate Keap1 expression in CSE-treated cells, which limits our ability to directly link our in vitro and in vivo findings. Future research could address this by building on evidence that DMF stabilizes Nrf2 by modifying Keap1 in pulmonary emphysema. Finally, we did not perform differential immune cell counts or cytokine profiling in BALF, which restricts a detailed understanding of the anti-inflammatory mechanisms. Including these analyses in future studies would provide a more comprehensive view of our findings.

## 5. Conclusions

The findings of this study affirm the significance of fumaric acid esters, specifically MMF and DMF, as promising therapeutic agents for conditions characterized by oxidative stress and pulmonary inflammation. In the in vitro model, MMF exhibited a partial protective effect against CSE-induced cytotoxicity, likely attributable to its ability to regulate redox balance and decrease levels of ROS, nitrite, and peroxynitrite. The observed reduction in lipid peroxidation, as indicated by lower MDA levels, further supports MMF’s antioxidant activity and its role in stabilizing cellular membranes. In the in vivo elastase-induced emphysema model, DMF demonstrated substantial protective effects, including the preservation of alveolar morphology, maintenance of elastic fiber density, and reduction in macrophage infiltration. Additionally, DMF restored the expression of antioxidant enzymes such as SOD1 and HO-1 and modulated the activity of MMP-9, underscoring its impact on tissue remodeling and the pulmonary inflammatory response. The activation of the Nrf2/ARE pathway appears to be a key mechanism through which fumarates exert these protective effects, enhancing the expression of antioxidant genes and mitigating oxidative stress in lung cells. This study provides robust evidence of the therapeutic potential of DMF in the context of inflammatory lung diseases, thereby extending the clinical relevance of a compound already successfully utilized in the treatment of multiple sclerosis and psoriasis. Given the short half-life of its main active metabolite, MMF, further research is warranted to optimize dosing strategies and fully elucidate the molecular pathways that drive its clinical efficacy in these new indications. These findings lay the groundwork for future investigations into the modulation of the Nrf2/NF-κB axis and the interplay between fumarates and metalloproteinase inhibitors, aiming to develop more effective therapeutic strategies for managing pulmonary emphysema and other chronic lung diseases.

## Data Availability

Data will be made available on request.

## Supplementary Materials

The following supporting information can be downloaded at: https://www.mdpi.com/article/10.3390/antiox14091141/s1, Figure S1: PC-9 viability (MTT) and lactate dehydrogenase (LDH) release in cells exposed to cigarette smoke extract (CSE) and treated with monomethyl fumarate (MMF) or the vehicle dimethyl sulfoxide (DMSO). Figure S2: Representative images of mouse lung tissue stained with Giemsa for visualization and quantification of mononuclear cells. Figure S3: Representative images of mouse lung tissue stained with Giemsa for visualization and quantification of mononuclear cells. Figure S4: Uncropped and unedited western blot membrane for PNK. Figure S5: Uncropped and unedited western blot membrane for SOD1. Figure S6: Uncropped and unedited western blot membrane for HO-1. Figure S7: Uncropped and unedited western blot membrane for MMP-9. Figure S8: Uncropped and unedited western blot membrane for PNK.

## Abbreviations

The following abbreviations are used in this manuscript:

APF	Aminophenyl fluorescein
ARE	Antioxidant response element
BALF	Bronchoalveolar lavage fluid
BSA	Bovine serum albumin
COPD	Chronic obstructive pulmonary disease
CSE	Cigarette smoke extract
DAB	3,3′-Diaminobenzidine
DCF	Dichlorofluorescein
DMF	Dimethyl fumarate
DNA	Deoxyribonucleic acid
FBS	Fetal bovine serum
GSTA2	Glutathione S-transferase A2
H&E	Hematoxylin and eosin
HBSS	Hanks’ balanced salt solution
HO-1	Heme oxygenase-1
Keap1	Kelch-like ECH-associated protein 1
LDH	Lactate dehydrogenase
Lm	Mean linear intercept
MDA	Malondialdehyde
MMF	Monomethyl fumarate
MMP-9	Matrix metalloproteinase 9
MTT	3-(4,5-dimethylthiazol-2-yl)-2,5-diphenyltetrazolium bromide
NAD+	Nicotinamide adenine dinucleotide (oxidized form)
NADH	Nicotinamide adenine dinucleotide (reduced form)
NF-κB	Nuclear factor kappa B
NQO1	NAD(P)H quinone dehydrogenase 1
Nrf2	Nuclear factor erythroid 2–related factor 2
PBS	Phosphate-buffered saline
PNK	Nitrotyrosine
PPE	Porcine pancreatic elastase
RPMI	Roswell Park Memorial Institute medium
ROS	Reactive oxygen species
RSN	Reactive species of nitrogen
SOD1	Superoxide dismutase 1
TBARS	Thiobarbituric acid reactive substances
TBS	Tris-buffered saline
Vv	Volume density
