# Supplementary material for: Modulation of Pulmonary Inflammation and the Redox Pathway In Vitro and In Vivo by Fumaric Ester
Source: Antioxidants (Basel). 2025 Sep 22;14(9):1141. doi: 10.3390/antiox14091141 (PMC12466633; doi:10.3390/antiox14091141)
Supplement: Supplementary file 1 [file antioxidants-14-01141-s001.zip › antioxidants-3818014-supplementary.docx]

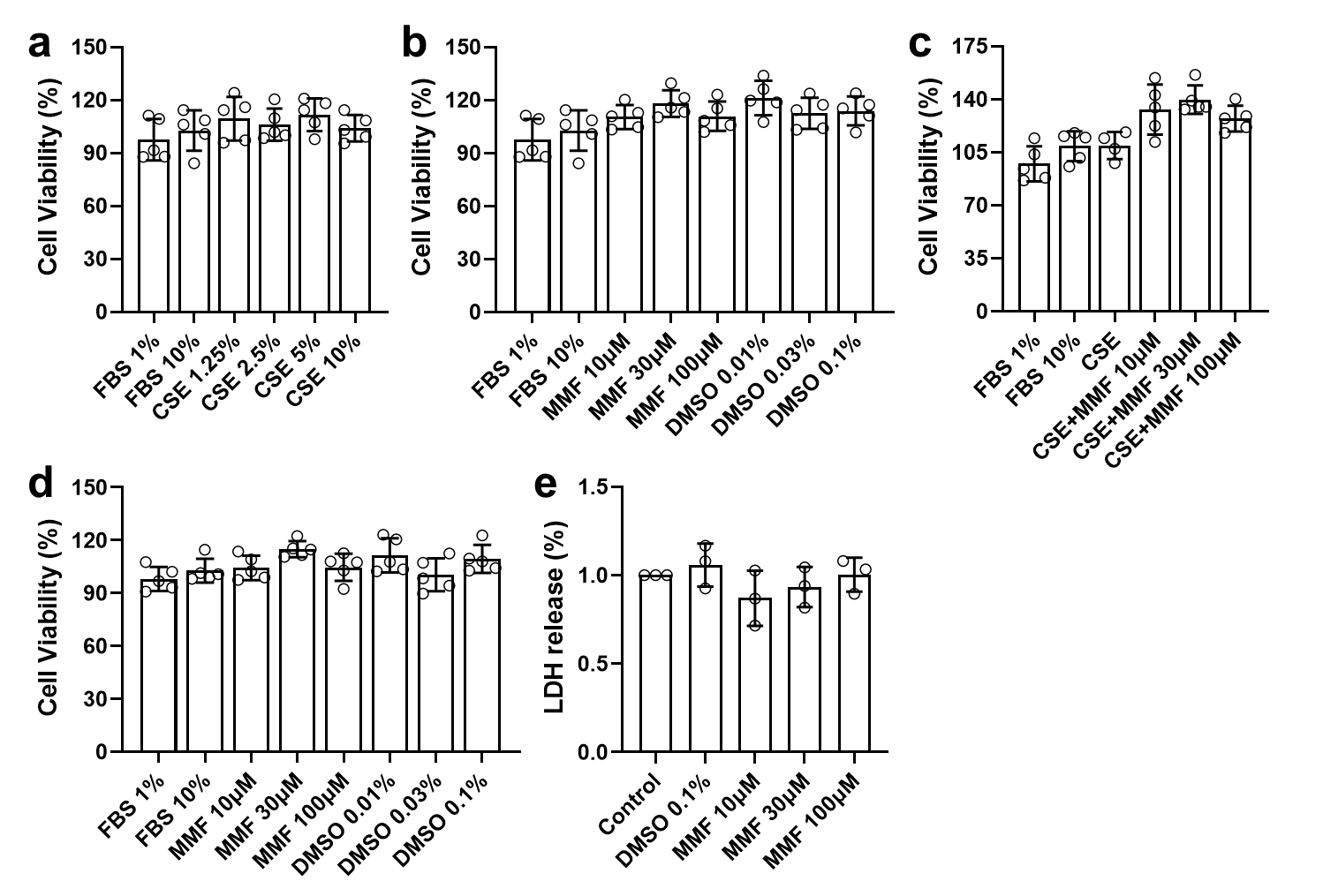


**Figure S1.** PC-9 viability (MTT) and lactate dehydrogenase (LDH) release in cells exposed to cigarette smoke extract (CSE) and treated with monomethyl fumarate (MMF) or the vehicle dimethyl sulfoxide (DMSO). Experimental procedures from a-c was performed during 1 h of incubation. Experimental procedures from d-e was performed during 24 h of incubation. (a) PC-9 viability of cells exposed to different concentrations of CSE (1.25%, 2.5%, 5%, and 10%); (b) PC-9 viability of cells treated with dimethyl fumarate (DMF) (10 µM, 30 µM, and 100 µM) or DMSO (0.01%, 0.03%, and 0.1%); (c) PC-9 viability of cells treated with DMF (10 µM, 30 µM, and 100 µM) in the presence of 10% CSE; (d) PC-9 viability of cells treated with DMF (10 µM, 30 µM, and 100 µM) or DMSO (0.01%, 0.03%, and 0.1%); (e) LDH release in cells treated with DMF (10 µM, 30 µM, and 100 µM) or DMSO (0.1%). Data are expressed as mean ± standard deviation from independent experiments. Statistical analysis was performed by using one-way ANOVA with Tukey’s post hoc test. The sample size for each column in the graphs is indicated by open circles.


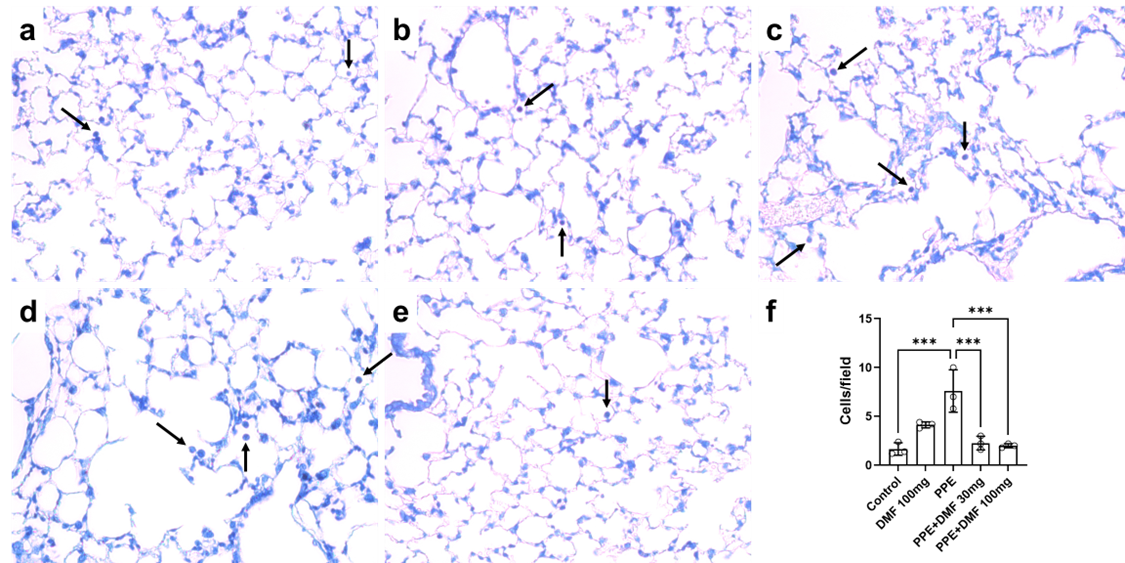


**Figure S2**. Representative images of mouse lung tissue stained with Giemsa for visualization and quantification of mononuclear cells. Arrows indicate examples of mononuclear cells. (a) Control group; (b) Control group receiving 100 mg/kg of dimethyl fumarate (DMF) orally; (c) Emphysema group instilled with porcine pancreatic elastase (PPE) at a concentration of 3U/mouse; (d) PPE-instilled group treated concomitantly with DMF at a dose of 30 mg/kg; (e) PPE-instilled group treated concomitantly with DMF at a dose of 100 mg/kg; (f) Quantification of mononuclear cells in mouse lung tissue by morphometry. Data are expressed as mean ± standard deviation from independent experiments. Statistical analysis was performed by using one-way ANOVA with Tukey’s post hoc test. ***p < 0.001. The sample size for each column in the graphs is indicated by open circles.


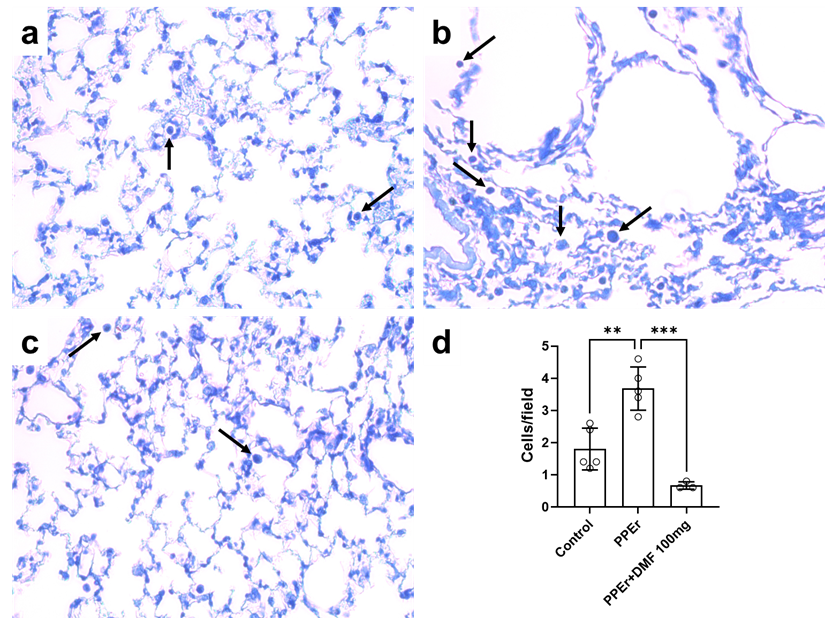


**Figure S3**. Representative images of mouse lung tissue stained with Giemsa for visualization and quantification of mononuclear cells. Arrows indicate examples of mononuclear cells. (a) Control group; (b) Emphysema group instilled with porcine pancreatic elastase (PPE) at a concentration of 3U/mouse and maintained until 65 days; (c) PPE-instilled group treated with DMF at a dose of 100 mg/kg. Treatment started at day 33; (d) Quantification of mononuclear cells in mouse lung tissue by morphometry. Data are expressed as mean ± standard deviation from independent experiments. Statistical analysis was performed by using one-way ANOVA with Tukey’s post hoc test. **p < 0.01 and ***p < 0.001. The sample size for each column in the graphs is indicated by open circles.


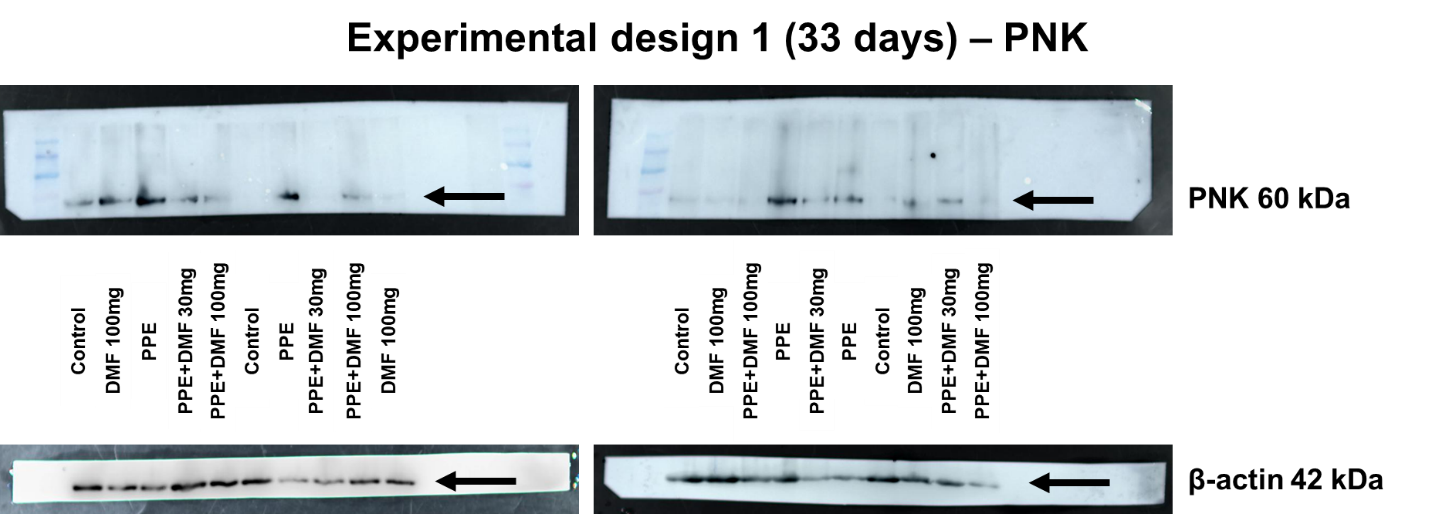


**Figure S4**. Uncropped and unedited western blot membrane for PNK.


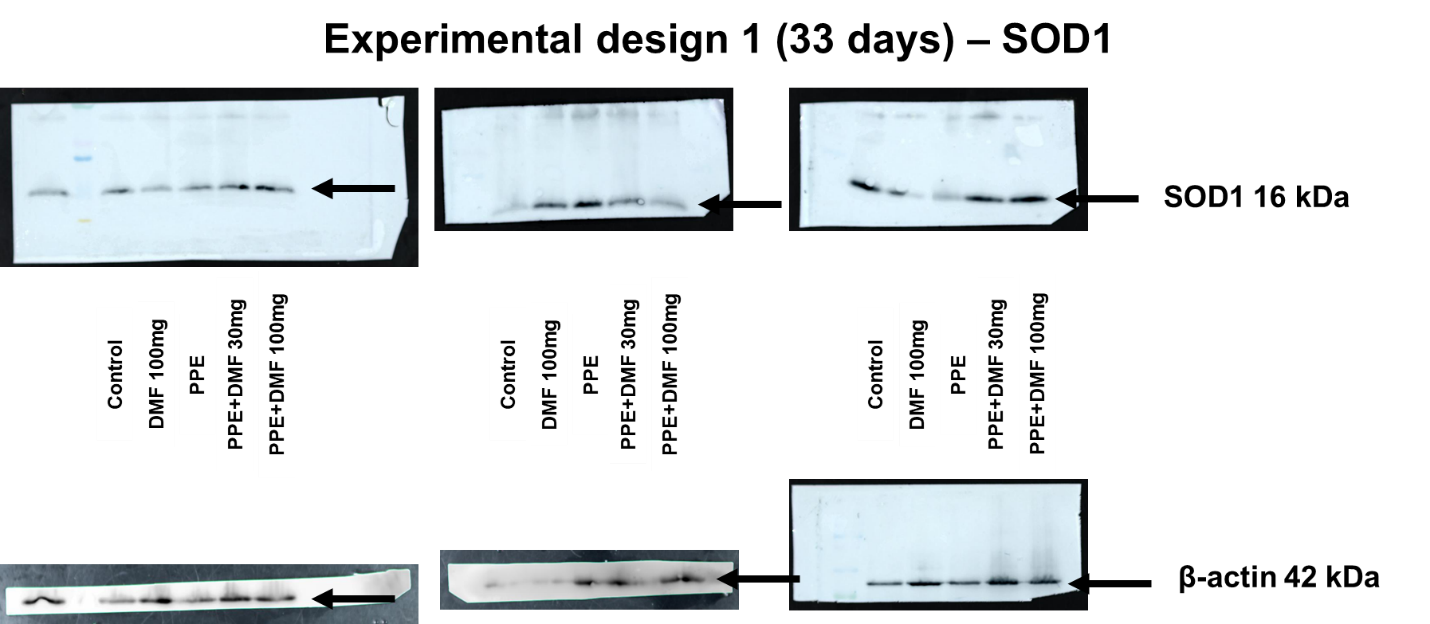


**Figure S5**. Uncropped and unedited western blot membrane for SOD1.


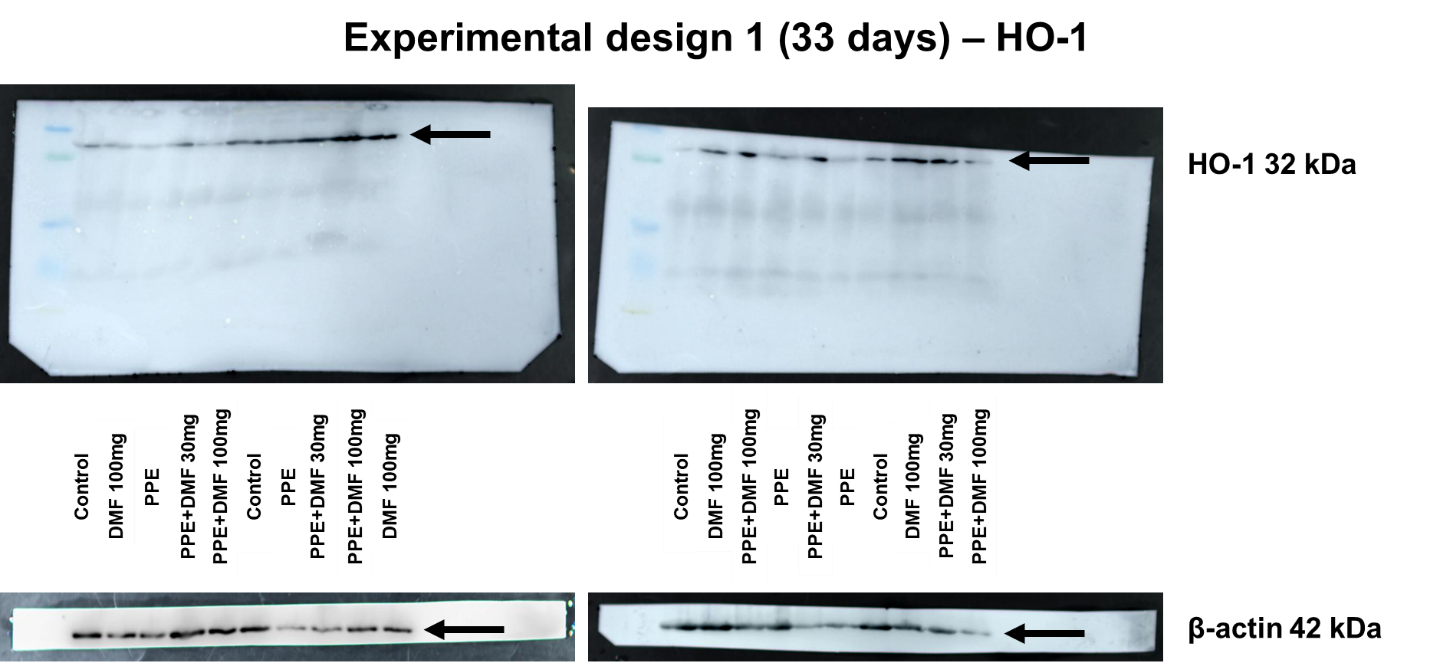


**Figure S6**. Uncropped and unedited western blot membrane for HO-1.


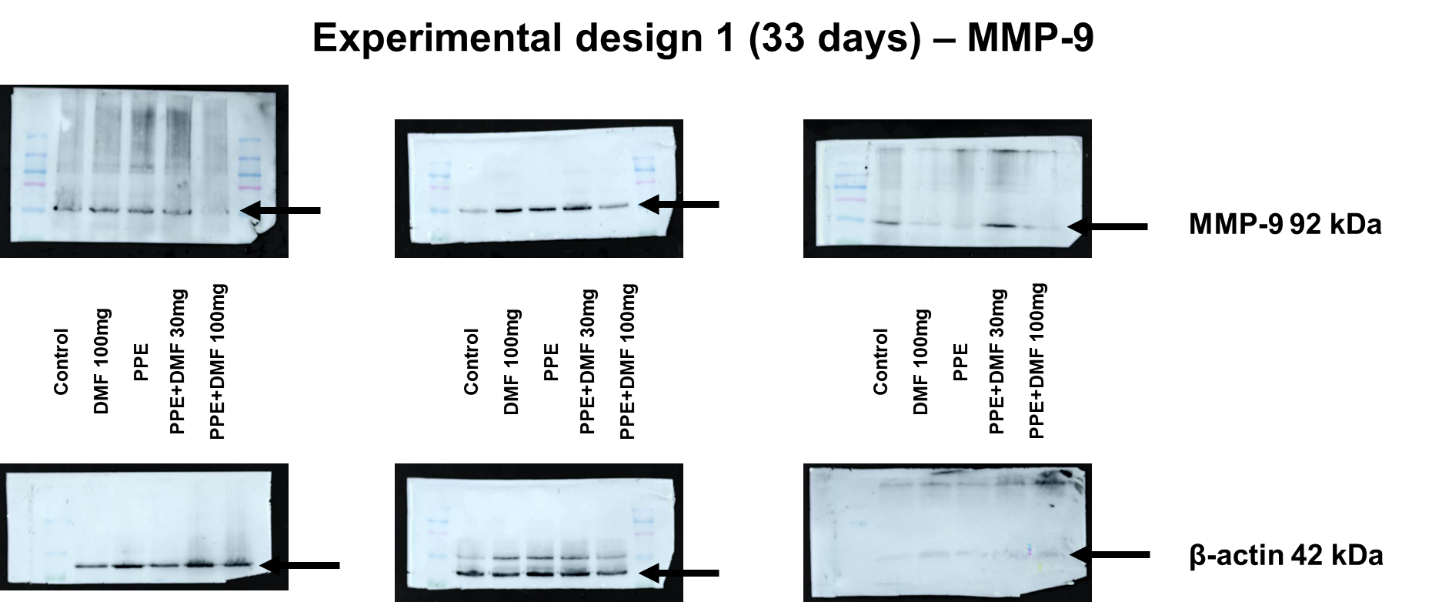


**Figure S7**. Uncropped and unedited western blot membrane for MMP-9.


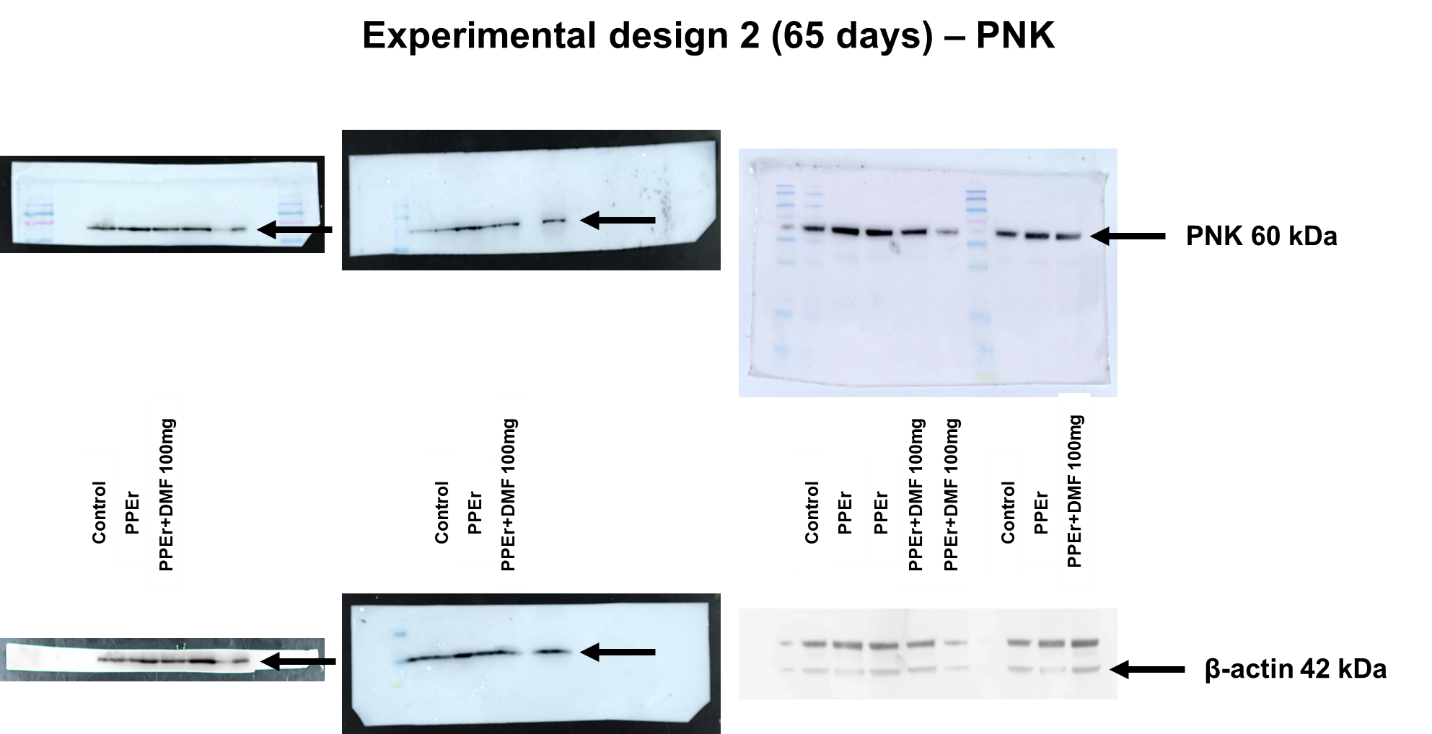


**Figure S8.** Uncropped and unedited western blot membrane for PNK.
